# Estimated collective effective dose to the population from nuclear medicine examinations in Slovenia

**DOI:** 10.2478/raon-2013-0048

**Published:** 2013-07-30

**Authors:** Damijan Skrk, Dejan Zontar

**Affiliations:** 1 Slovenian Radiation Protection Administration, Ljubljana, Slovenia; 2 “Jožef Stefan” Institute, Ljubljana, Slovenia

**Keywords:** nuclear medicine, diagnostic procedures, collective effective dose, population exposure, dose per capita

## Abstract

**Background:**

A national survey of patient exposure from nuclear medicine diagnostic procedures was performed by Slovenian Radiation Protection Administration in order to estimate their contribution to the collective effective dose to the population of Slovenia.

**Methods:**

A set of 36 examinations with the highest contributions to the collective effective dose was identified. Data about frequencies and average administered activities of radioisotopes used for those examinations were collected from all nuclear medicine departments in Slovenia. A collective effective dose to the population and an effective dose per capita were estimated from the collected data using dose conversion factors.

**Results:**

The total collective effective dose to the population from nuclear medicine diagnostic procedures in 2011 was estimated to 102 manSv, giving an effective dose per capita of 0.05 mSv.

**Conclusions:**

The comparison of results of this study with studies performed in other countries indicates that the nuclear medicine providers in Slovenia are well aware of the importance of patient protection measures and of optimisation of procedures.

## Introduction

The exposure of the global population to ionizing radiation is rising rapidly, nearly exclusively due to increasing medical use of radiation. Diagnostic X-rays is by far the largest source of medical exposure in most developed countries, while the contribution of nuclear medicine examinations is between 4% and 14%.^1^ For that reason also legislation of European Union requires that the Member States determine the distribution of individual doses from medical exposure for their population.^2^,^3^

Based on findings of the “DOSE DATAMED” project European Commission prepared and published European Guidance on Estimating Population Doses from Medical X-ray Procedures in 2008.^1^ In the beginning of 2011 a follow-up project, named “Study on European Population Doses From Medical Exposure”, or “DOSE DATAMED2” (DDM2) was launched. The objective of the DDM2 project is to collect available data on the patient doses from the radiodiagnostic procedures in the European Union.

An estimate of doses to patients from nuclear medicine examinations in Slovenia as a whole has not been carried out previously. Therefore in 2011 Slovenian Radiation Protection Administration (SRPA) performed a survey about the population exposure from nuclear medicine procedures. This article summarizes the results of the survey and shows the impact of nuclear medicine examinations on the population dose.

## Materials and methods

In order to assess population exposure from nuclear medicine in terms of the collective or per capita effective dose, it is necessary to estimate frequency and mean effective dose for each type of examination that makes a significant contribution to the annual collective effective dose in a country. At the European level a set of 28 diagnostic procedures that are contributing most significantly to the collective effective dose (Table 1) were identified by DDM2 project.^3^

All nuclear medicine departments in Slovenia were asked to report their annual workload and the average administered activity of the radiopharmaceutical for the 28 nuclear medicine procedures. Data about additional examinations that were not included in the list but are frequently performed at their departments were also requested. All requested data were received from all nuclear medicine departments in Slovenia. Additional 20 examinations were reported together with the relevant data.

Among the 28 examinations listed by the DDM2 project, myocardial perfusion (PET) with radioisotope ^15^O and dopamine transporter imaging (parkinsonism) with radioisotope ^123^I in chemical form β-CIT were not performed in Slovenia. The final analysis thus included 26 examinations proposed by the DDM2 project and additional 20 procedures that made a significant contribution to the collective effective dose. Ten out of the 20 additional examinations identified during the project contribute less than 0.5% to the total collective dose and are consequently not presented in Table 2, while the ten most relevant ones are.

The collected data were verified by comparing them to information available from licensing and inspection procedures and with the amounts of imported radiopharmaceuticals. Results from all those approaches were found to be consistent.

Mean activity per examination in Slovenia was derived from the mean activities as reported from each department, weighted by relative number of examinations performed at any given department.

The typical effective dose per nuclear medicine examination was estimated by multiplying mean activity administered per examination with a conversion factor (mSv/MBq) as given in product specifications of radiopharmaceuticals or as published by International Commission on Radiological Protection (ICRP). Relevant publications are ICRP 53^4^ and its updated and extended editions ICRP 80^5^ and ICRP 106.^6^ Conversion factors for examinations listed in Table 1 are taken from ICRP publications, while in Table 2 product specifications of radiopharmaceuticals provided by nuclear medicine departments are used. The collective effective dose from each examination was calculated by multiplying the number of performed examinations with the typical effective dose and the total collective effective dose is a sum of collective effective doses for each examination.

The effective dose per capita was calculated by dividing the total collective effective dose with the total population of Slovenia that was taken as 2.05 million (end of 2011 data).^7^

Obtaining information from all nuclear medicine departments ensures a representative data sample. Thereby the major source of error is uncertainties in the number of performed examinations as reported from nuclear medicine departments. Uncertainties of the results will be further elaborated in the next section.

## Results

Based on the gathered data, the analysis of the 36 nuclear medicine examinations was performed in order to estimate the typical effective dose of each type of examination as well as the collective dose of each group of examinations and their contribution to the total collective dose. In addition, relative contributions of different radioisotopes and workloads of different departments are presented.

Data about the 26 nuclear medicine examinations as selected by the DDM2 project are presented in Table 1 and in Table 2 the additional ten examinations are listed. The first three columns list types of examinations with radionuclide and chemical form of radiopharmaceutical used. The next four columns list number of examinations performed yearly, average administered activity per examination with the range of average activities (in brackets), conversion factors and the effective dose per examination as calculated from the listed data. In the last column contributions of each examination to the collective effective dose from nuclear medicine procedures in Slovenia in 2011 are presented.

The remaining ten examinations (a total of 1170 examinations performed in 2011) are not listed as they contribute less than 0.5% (0.41 manSv) to the total collective effective dose from nuclear medicine procedures.

Five examinations with the highest contribution to the total collective effective dose (bone imaging and four myocardial procedures) contribute nearly 64%, while the top ten examinations contribute 85% and top twenty examinations contribute 96%. Based on the collected data that include 31,187 nuclear medicine examinations, the collective effective dose from nuclear medicine procedures in 2011 was estimated to be 102 manSv, *i.e*. 0.05 mSv per inhabitant.

**FIGURE 1.**
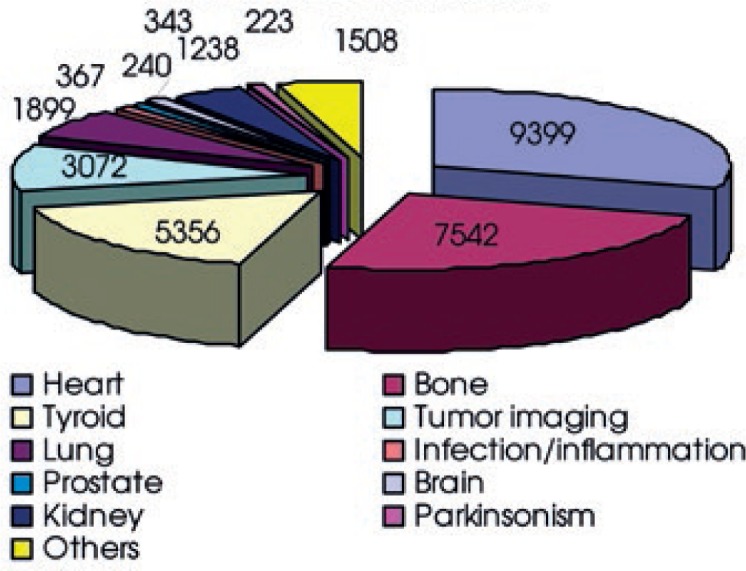
Number of nuclear medicine examinations grouped according to the organ, target or closely similar objectives.

**FIGURE 2.**
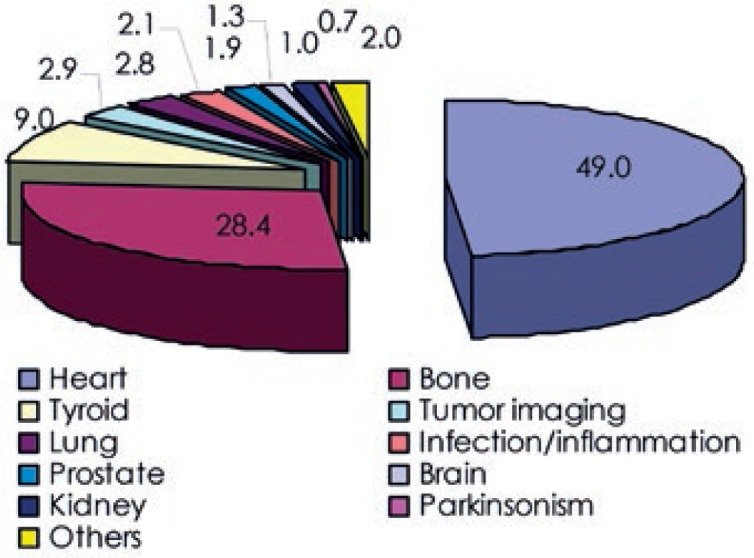
Collective effective dose from nuclear medicine examinations grouped according to the organ, target or closely similar objectives.

To determine contributions to the total collective dose from different examination groups, examinations were grouped together according to the organ, target or closely similar objectives. Examinations were grouped into the following categories: heart, bone, tyroid, lung, prostate, brain, kidney, infection/inflammation, tumor imaging and parkinsonism. Bone scans, heart and tyroid examinations contribute more than 70% to the total annual number of nuclear medicine examinations and over 85% to the total collective dose. Numbers of nuclear medicine examinations in different categories are shown in Figure 1 and their contributions to the total collective dose in Figure 2.

**FIGURE 3.**
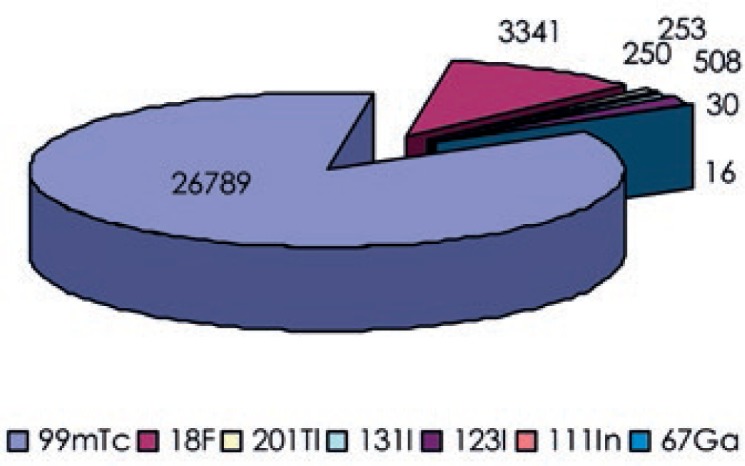
Number of nuclear medicine examinations according to the isotope used.

**FIGURE 4.**
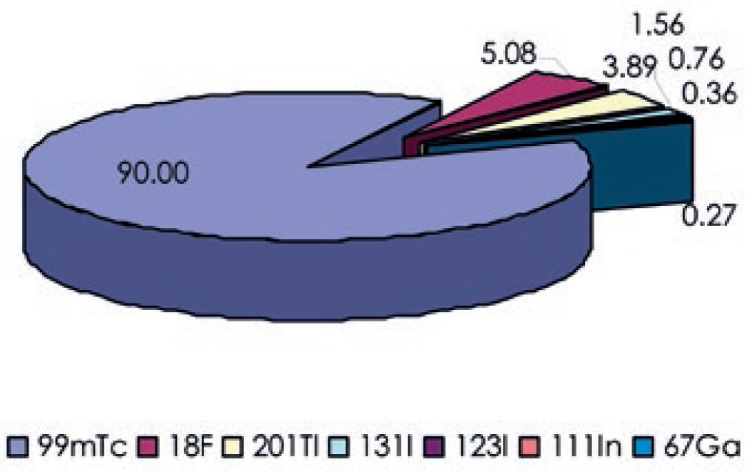
Collective effective dose from nuclear medicine examinations according to the isotope used.

In Slovenian nuclear medicine departments the following isotopes are used for diagnostic purposes: ^99m^Tc, ^18^F, ^201^Tl, ^131^I, ^123^I, ^111^In and ^67^Ga. Isotope ^99m^Tc is used in all nuclear medicine departments, ^123^I is used in three, ^18^F, ^111^In and ^67^Ga in two, while ^201^Tl and ^131^I are used in only one department each. Most procedures were performed with ^99m^Tc and ^18^F, with ^99m^Tc being used in 86% and ^18^F in 11% of all procedures. The full list is shown in Figure 3 and their contributions to total collective effective dose from nuclear medicine examinations are shown in Figure 4. By far the largest contribution to the collective effective dose from nuclear medicine examinations is from the use of ^99m^Tc (90%) and radiopharmaceuticals with isotope ^18^F contribute another 5%. Radiopharmaceuticals with ^111^In and ^67^Ga have almost negligible contribution to the collective dose. The contributions are expected to change in the future as the number of PET examinations is expected to increase and use of ^201^Tl radiopharmaceuticals to decrease.

**FIGURE 5.**
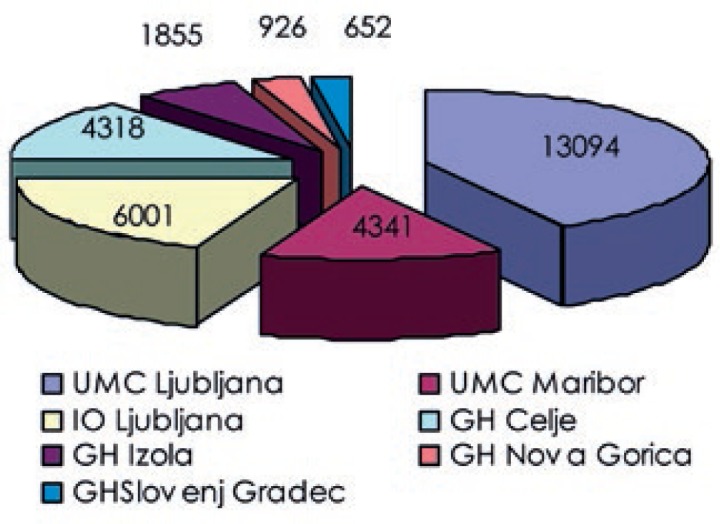
Number of examinations per nuclear medicine department.

**FIGURE 6.**
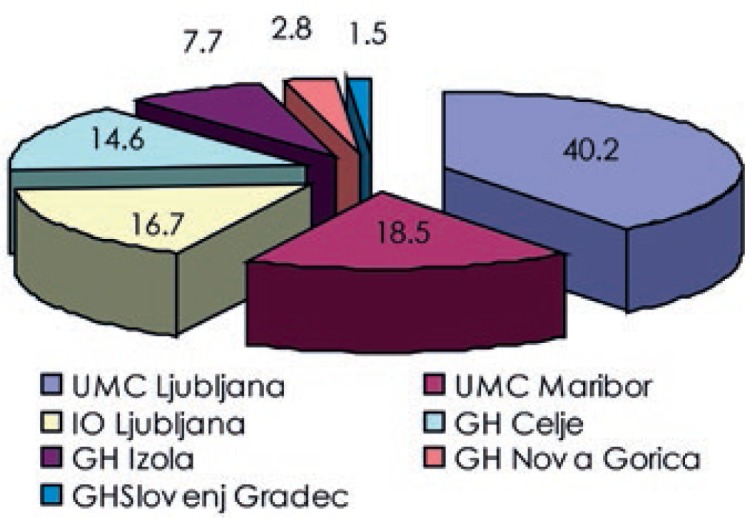
Collective effective dose (manSv) per nuclear medicine department.

The analysis of the workload distribution among the departments showed that nearly 42% of all examinations were performed in Nuclear medicine department of the University Medical Center Ljubljana. Nuclear medicine departments of the University Medical Center Maribor, the Institute of Oncology Ljubljana and General hospital Celje, with comparable workloads, contributed together close to 47%, with the remaining three departments accounting for less than 12%. Numbers of nuclear medicine examinations per department are shown in Figure 5 and their contributions to the total collective dose in Figure 6.

### Uncertainties of the results

The collected data are a representative sample of nuclear medicine practice in Slovenia. Analysed procedures were estimated to contribute over 99% to the total collective effective dose from diagnostic procedures in nuclear medicine in Slovenia.

The only recognized source of uncertainty that could considerably influence the reliability of the results was uncertainties in the number of performed examinations. Information from two departments which reported exact values were assumed to be very precise as they were extracted from the local databases. Uncertainties on total number of procedures from four departments which reported rounded values were conservatively estimated to be 3% to 15%, while uncertainty of values scaled to the yearly level from one department was estimated to 8%. The total uncertainty estimation took into account relative contributions and conservative assumptions of uncertainties for each department. Under such assumptions the total uncertainty on the collective effective dose was estimated to be less than 4% (CL 95%). Possible uncertainties from incorrect matching of procedures and incorrect reported mean activities per examination were checked for by comparison with independent data from official SRPA records. Based on the findings their contribution was assumed to be negligible.

## Discussion

In Slovenia the collective effective dose to the population from radiological examinations is not being estimated regularly. The presented survey is the first attempt to gather data about all nuclear medicine examinations performed in all departments in order to estimate their contribution to the total collective effective dose. Gathered data also enable the comparison of the frequencies and the annual per capita doses from medical nuclear medicine examinations in Slovenia with other countries.^1^ In the future, gathered data will enable observation of trends in the frequencies, annual collective dose and/or per capita dose from nuclear medicine examinations in Slovenia.

The total annual number of nuclear medicine examinations per 1000 inhabitants in Slovenia in 2011 was found to be 15 and the average annual effective dose per capita was 0.05 mSv. Taking into account an unpublished SRPA study of radiological procedures, the contribution of nuclear medicine examinations to the collective effective dose from diagnostic medical exposures was 7%, while in European countries it is between 4% and 14%.^1^ The total annual number of nuclear medicine examinations per 1000 inhabitants in European countries ranges from 8 to 56. The number of nuclear medicine examinations per 1000 population in Slovenia is lower compared to Belgium (56), Germany (42), Luxemburg (38) and Netherlands (18) and slightly higher than in Switzerland (13), Norway (12), Sweden (12), Untied Kingdom (11) and Finland (8)^(1,8)^. As a result of a lower annual number of nuclear medicine examinations as well due to optimised levels of administered activities of radioisotopes per examination, the level of annual effective dose per capita in Slovenia is relatively low compared to European average.

The total average annual effective dose from nuclear medicine procedures per capita in European countries ranges from 0.03 mSv to 0.20 mSv. The average annual effective dose in Slovenia is lower compared to Belgium (0.20 mSv, 1999), Luxembourg (0.16 mSv, 2002), Germany (0.11 mSv, 2002), Netherlands (0.07 mSv, 2002), Switzerland (0.07 mSv, 2004), the same as in Norway (0.05 mSv, 2004) and higher than in Sweden (0.04 mSv, 2005), United Kingdom (0.03 mSv, 2004) and in Finland (0.03 mSv, 2009).^(1,8)^

Bone scans and heart examinations were most frequent procedures both in Slovenia and in other countries for which authors had available data. A higher number of tyroid examination in Slovenia is due to the iodine deficiency, the same reason as for its higher contribution in Germany and Luxemburg.

Average ratio between maximal and minimal average administered activity between departments for any particular examination was found to be 1.4, which seems not to be a substantial difference^1^. Nevertheless, factors such as poor performance of imaging instrumentation and procedure parameters that influence the image quality and may result in higher average administered activities should be carefully investigated in close collaboration with the professional bodies in the nuclear medicine field. In addition special attention should be given to nuclear medicine examinations causing higher patient exposures, especially to those which are performed frequently. Evaluation of new available radiopharmaceuticals offering better performance or causing lower patient exposure should be performed regularly.

Frequent updates of information about nuclear medicine practice are essential as rapid technological development, new techniques and instrumentation as well as new procedures would affect both administered activity and frequency of particular examinations. Although the resources required to perform such surveys and analyses are considerable, it is proposed that they should be repeated at least every 5 years.^1^

## Conclusions

In Slovenia the total collective effective dose from nuclear medicine procedures was estimated to be 102 manSv in 2011, while the estimated effective dose per inhabitant was 0.05 mSv. This presents 7% of the total collective dose from all medical exposure examinations.

Nearly half of the collective effective dose from nuclear medicine examinations was caused by heart examinations. It is shown that procedures with ^99m^Tc were the major source of the total collective dose from nuclear medicine procedures, contributing almost 90%, while combined contribution of ^131^I, ^123^I, ^111^In and ^67^Ga procedures was less than 3%. The contribution of PET examinations was 5% in 2011 and is anticipated to increase in the future.

Presented results show that the nuclear medicine practice in Slovenia is close to the level of European countries where the awareness of patient protection and need for optimisation procedures is assumed to be high. Nevertheless, there is always room for improvement and optimisation. Regularly updating written procedures for examinations and establishment of national diagnostic reference levels in close cooperation with nuclear medicine professional bodies would be recommended.

Due to a rapid technological development, surveys and analysis of the doses from medical exposure procedures should be performed regularly. Availability of updated information will enable the proper optimisation process of nuclear medicine procedures and the adequate decision-making in the field of radiation protection on the national as well as on the international level in the future.

## Figures and Tables

**TABLE 1.** Data about the 26 nuclear medicine examinations as selected by the Study on European Population Doses From Medical Exposure (DDM2) project

**Examination**	**Radionuclide**	**Chemical form**	**Number of examinations**	**Mean activity (MBq) per examination (min-max)**	**Conversion factor (mSv/MBq)**	**Effecitve dose per examination(mSv)**	**Collective effective dose (manSv)**
Bone imaging	^99m^Tc	Phosphates, phosphonates	7542	683 (550–700)	5.70E-03^5^	3.89	29.36
Myocardial perfusion, rest	^99m^Tc	Tetrofosmin	2700	591 (520–600)	7.60E-03^5^	4.49	12.13
Myocardial perfusion, exercise	^99m^Tc	MIBI	1936	583 (520–630)	7.90E-03^5^	4.61	8.92
Myocardial perfusion, rest	^99m^Tc	MIBI	1577	582 (520–630)	9.00E-03^5^	5.24	8.26
Thyroid imaging (oral administration, no blocking)	^99m^Tc	Pertechnetate	4546	97 (74–120)	1.30E-02^5^	1.26	5.73
Myocardial perfusion, exercise	^99m^Tc	Tetrofosmin	1370	580 (520–600)	7.00E-03^5^	4.06	5.56
MUGA, cardiac blood pool, flow (equilibrium)	^99m^Tc	Tc-labelled erythrocytes	608	923 (740–925)	7.00E-03^5^	6.46	3.93
Myocardial perfusion	^201^Tl	Chloride	250	111 (111–111)	1.40E-01^6^	15.54	3.89
Lung perfusion	^99m^Tc	MAA	1518	143 (115–185)	1.10E-02^5^	1.57	2.38
Infection/inflammation imaging	^99m^Tc	labbelled white blood cells	303	483 (450–650)	1.10E-02^5^	5.31	1.61
Parathyroid imaging	^99m^Tc	MIBI	279	624 (444–777)	9.00E-03^5^	5.61	1.57
Thyroid metastases (after ablation, uptake 0%)	^131^I	Iodide	118	148 (148–148)	6.10E-02^4^	9.03	1.07
Tumor imaging (PET)	^18^F	FDG	1350	370 (370–370)	1.90E-03^4^	0.70	0.95
Renal imaging	^99m^Tc	MAG 3	1126	116 (100–185)	7.00E-03^5^	0.81	0.92
Dopamine transporter imaging (parkinsonism)	^123^I	Ioflupane (DaTscan)	223	121 (115–125)	2.40E-02^6^	2.89	0.65
Neuroendocrine tumors/somatostatin receptors imaging	^111^In	Pentetreotide (OctreoScan)	30	222 (222–222)	5.40E-02^5^	11.99	0.36
Infection/inflammation imaging	^67^Ga	Gallium citrate	16	177 (120–200)	1.00E-01^5^	17.68	0.27
Infection/inflammation imaging	^99m^Tc	Monoclonal antibody (LeucoScan)	48	553 (550–555)	8.00E-03^6^	4.42	0.21
Myocardial perfusion (PET)	^18^F	FDG	30	300 (300–300)	1.90E-02^4^	5.70	0.17
Cerebral blood flow	^99m^Tc	ECD (Neurolite)	119	657 (555–700)	2.20E-03^6^	1.45	0.17
Cerebral blood flow	^99m^Tc	Exametazime (HMPAO,Ceretec)	22	700 (700–700)	9.30E-03^5^	6.51	0.14
Thyroid imaging (thyroid uptake 35%)	^123^I	Iodide	278	15 (15–15)	2.20E-02^5^	0.34	0.09
MUGA, cardiac blood pool, flow (equilibrium)	^99m^Tc	DTPA	20	740 (740–740)	4.90E-03^5^	3.63	0.07
Renal imaging	^99m^Tc	DTPA	80	185 (185–185)	4.90E-03^5^	0.91	0.07
Tumor imaging (PET) + Diagnostic CT	^18^F	FDG	81	370 (370–370)	1.90E-03^5^	0.70	0.06
Renal imaging	^99m^Tc	DMSA	32	91 (80–100)	8.80E-03^5^	0.80	0.03

**Total**			**26202**				**88.57**

DMSA = dimercaptosuccinic acid; DTPA = diethylene triamine pentaacetic acid; ECD = Neurolite; FDG = Fluorodeoxyglucose (^18^F); MAA = technetium ^99m^Tc albumin aggregated; MIBI = technetium (^99m^Tc) sestamibi; MUGA = multi gated acquisition scan

**TABLE 2.** Data about the 10 additional nuclear medicine examinations with the highest contribution to the collective effective dose to the population from nuclear medicine procedures in Slovenia

**Examination**	**Radionuclide**	**Chemical form**	**Number of examinations**	**Mean activity (MBq) per examination (min-max)**	**Conversion factor (mSv/MBq)**	**Effecitve dose per examination (mSv)**	**Collective effective dose (manSv)**
Myocardial perfusion, persantin	^99m^Tc	Tetrofosmin	908	600 (600–600)	1.12E-02	6.72	6.10
Prostate imaging (PET)	^18^F	Choline	240	250 (250–250)	3.13E-02	7.83	1.88
Tumor imaging (PET) + Low dose CT	^18^F	FDG	1437	370 (370–370)	1.90E-03	0.70	1.01
Brain imaging (PET)	^18^F	FDG	203	250 (250–250)	2.00E-02	5.00	1.01
Labelled erythrocytes	^99m^Tc	Tc-labelled erythrocytes	96	569 (450–600)	1.40E-02	7.97	0.76
Neuroendocrine tumors/somatostatin receptors imaging	^99m^Tc	EDDA/HYNIC-TOC	174	600 (600–600)	5.00E-03	3.00	0.52
Pre-ablation thyroid remnant imaging (oral administration, no blocking)	^131^I	Iodide	135	4 (4–4)	1.00E+00	3.70	0.50
Liver hemangiom	^99m^Tc	Tc-labelled erythrocytes	66	450 (450–450)	1.40E-02	6.30	0.42
Lung ventilation	^99m^Tc	Tctechnegas	380	40 (40–40)	2.43E-02	0.97	0.37
Sentinel node	^99m^Tc	Nanocoll	176	124 (74–133)	1.70E-02	2.11	0.37

Total			3815				12.94

EDDA/HYNIC-TOC = EDDA/HYNIC-Tyr3-octreotide; FDG = Fluorodeoxyglucose (^18^F)
